# Efficacy comparisons of enteral nutrition and parenteral nutrition in patients with severe acute pancreatitis: a meta-analysis from randomized controlled trials

**DOI:** 10.1042/BSR20181515

**Published:** 2018-11-16

**Authors:** Ping Wu, Liang Li, Weijia Sun

**Affiliations:** 1 Department of Social Medicine and Health Management, Xiangya School of Public Health, Central South University, Changsha, Hunan Province 410078 China; 2 Department of Science and Education, Peace Hospital of Changzhi Medical College, Changzhi, Shanxi Province 046000, China; 3 Department of Medical Services, Xiangya Hospital, Central South University, Changsha 410008, Hunan Province, China; 4 Department of General Surgery, Xiangya Hospital, Central South University, Changsha 410008, Hunan Province, China

**Keywords:** Enteral nutrition, meta-analysis, parenteral nutrition, randomized controlled trial, severe acute pancreatitis

## Abstract

We conducted a comprehensive analysis to evaluate the treatment efficacy and safety of enteral nutrition (EN) and parenteral nutrition (PN) in severe acute pancreatitis (SAP) patients, and to provide a basis for their evidence based application in a clinical setting. We conducted a systematic online search of the PubMed, Web of Science, Wanfang, and China National Knowledge Infrastructure databases, from their inception to November 2017. Studies were subjected to further screening if they met the inclusion/exclusion criteria. Eleven studies were subjected to qualitative and quantitative synthesis; these included a total of 562 patients (281 for EN and 281 for PN). No significant heterogeneity across studies was found. The results indicated that EN can significantly decrease the mortality rate (relative risk [RR] = 0.43, 95% confidence interval [CI]: 0.23–0.78, *P*=0.006), and lowers the risk of infection and complications (RR = 0.53, 95% CI: 0.39–0.71, *P*=0.000) more so than does PN. The EN group had a similar risk of multiple organ failure (MOF) compared with the PN group (RR = 0.63, 95% CI: 0.39–1.02, *P*=0.059). The use of EN was also found to significantly reduce mean hospitalization time (mean difference = −2.93, 95% CI: −4.52–1.34, *P*=0.000). No publication bias was found. Our meta-analysis suggested that EN, but not PN, significantly reduced the risk of mortality, infection, and complications for patients with SAP. EN support also decreased the rate of MOF and surgical intervention. EN is recommended as an initial treatment option for patients with SAP.

## Introduction

Acute pancreatitis is a common abdominal disease that can be divided into mild and severe acute pancreatitis (SAP) [1]. The mortality rate of mild acute pancreatitis is very low. SAP is a hemorrhagic, necrotizing pancreatitis characterized by more severe symptoms, and may be accompanied by shock, viscera dysfunction, and severe metabolic derangement [2]. SAP progresses rapidly and mortality rate is high. SAP is often accompanied by systemic inflammatory response syndrome (SIRS), which is a serious inflammatory response that significantly increases catabolism and energy consumption [3]. This often results in rapid loss of reserve nutrients, imbalances in acid–base regulation, and loss of water and electrolytes in the body. These metabolic abnormalities, in conjunction with failure to receive timely treatment, may lead to multiple organ failure (MOF) involving the heart, lungs, and kidneys. This further worsens the prognosis and overall survival rate of patients with SAP [4,5]. Therefore, nutritional support is very important as a therapeutic measure for patients with SAP. The two most commonly used nutrition support therapies are enteral nutrition (EN) and parenteral nutrition (PN) [6,7]. Previous guidelines on the treatment of acute pancreatitis have stated that PN is the preferred option for management of SAP, despite the fact that long-term use of PN has been shown to cause systemic inflammatory responses and MOF. PN has also been shown to have a higher cost, confer an increased risk of catheter-related infection, and increase the rate of electrolyte imbalance [8]. The intestinal mucosal function in patients with EN can protect against damage and reduce the number of enterogenous infectious complications. However, in the process of administering EN, the patient may experience gastrointestinal discomfort, diarrhea, and abdominal distension [9]. Furthermore, PN does not stimulate pancreatic juice secretion, which can reduce the burden on the gastrointestinal tract, and may increase the overall incidence of enterogenous and catheter-related infections [10]. However, some studies have found that EN support for patients with SAP is similar to intestinal nutrition in terms of its ability to reduce mortality and infection rates. Conversely, some scholars have found that EN support therapy can stimulate pancreatic secretion and worsen pancreatitis, and therefore should be avoided in SAP patients with intestinal obstruction [10]. In the present study, we conducted a comprehensive analysis to evaluate the treatment efficacy and safety of EN and PN in SAP, and to provide an evidence base for their use in the clinical setting.

## Materials and methods

The present meta-analysis followed the guidelines set forth by the Preferred Reporting Items for Systematic reviews and Meta-analysis (PRISM) checklists [11]. No registered protocol was applied.

### Literature search

We conducted a systematic online search of the PubMed, Web of Science, Wanfang, and China National Knowledge Infrastructure databases, from their inception to November 2017. The following MeSH terms and keywords were used: SAP or acute necrotizing pancreatitis; EN or enteral feeding; PN or parenteral feeding. We also reviewed reference sections for further reviews and related studies. We restricted the search languages to English and Chinese.

### Criteria for inclusion and exclusion

Two authors independently performed the initial search, deleted duplicates, and screened the titles and abstracts. The study inclusion criteria were as follows: (1) design type: randomized controlled trial or cohort study; (2) study population: children or adults with SAP who required enteral or PN for at least 48 h; (3) intervention: enteral or PN. (4) Comparison: EN with PN. Exclusion criteria included (1) experimentation studies, comments, reviews, letters, and conferences abstracts, and (2) studies with very small sample sizes (*n*<30). In cases of continuing or duplicate studies, only the most recent data were used.

### Data extraction

Two authors independently performed the data extraction. Disagreements were resolved through consensus discussion. We extracted the following information from each study using a standardized Excel (Microsoft Corp., Redmond, WA, U.S.A.) sheet: first author, year of publication, mean age of EN and PN groups, sample size, sex distribution (male/female), and treatment duration. We also attempted to directly contact the corresponding author if there was any missing information. The primary outcome was mortality rate, while secondary outcomes included the infection and complication rates, surgical intervention, MOF, and mean hospitalization time.

### Quality assessment

We assessed the quality of each study using the guidelines set forth by the Cochrane Library [12]. The risk of bias tool was used to classify the studies as being at high risk, low risk, and uncertain risk of bias, for each of the following domains: selection bias (random sequence generation, allocation concealment), performance bias (blinding of participants and personnel), detection bias (blinding of outcome assessment), attrition bias (incomplete outcome data), and reporting bias (selective reporting). Studies with a high risk of bias in more than one domain were classified as high risk, while those with low bias risk in all domains were considered low risk. Any studies that did not fall into either of these two bias categories were classified as unclear risk.

### Statistical analysis

We used the Stata (Stata Corp, LP) and RevMan 5.3 (Cochrane library) programs to analyze the data. We used the relative risks (RRs) and their 95% confidence intervals (CIs) to assess the differences between EN and PN for binary data (mortality, infection and complication, surgical intervention, and MOF rates). We also calculated the weighted mean differences (MDs) and 95% CIs to evaluate differences in mean hospitalization time. Heterogeneity across studies was assessed by Chi-square analysis and the *I*^2^ statistic. An *I*^2^ > 50% indicated significant heterogeneity across studies [13]. In such cases, a random-effect model was used. Otherwise, a fixed-effect model was employed. Sensitivity analysis was conducted by excluding one study at a time, from the analysis. Publication bias was assessed using the Begg’s and Egger’s tests [14,15]. *P*<0.05 was considered to be significant.

## Results

### Study selection

**Figure 1:**
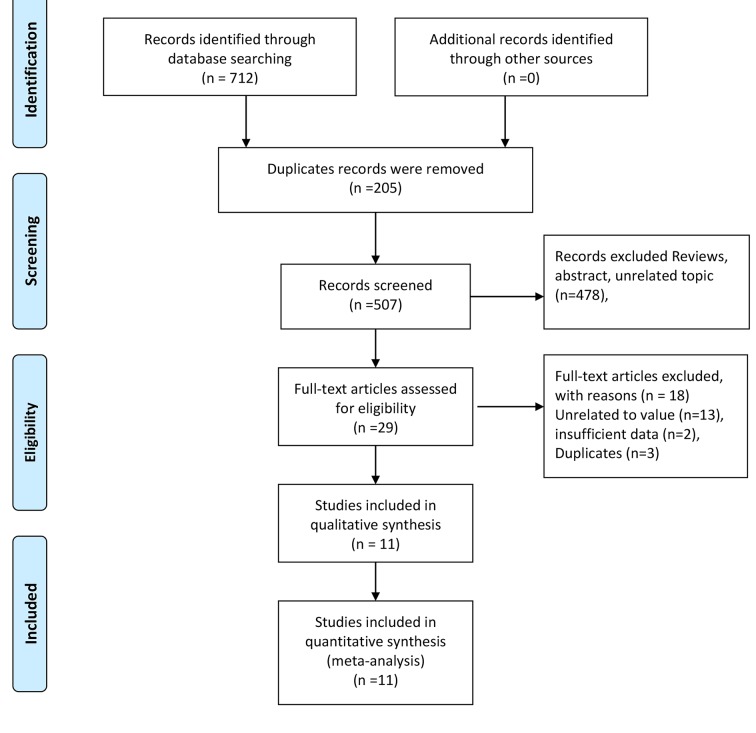
Flow chart of study selection

Figure 1 shows the flow of study screening and exclusion. We identified 712 studies in our database search; 205 duplicate records were removed. Thus, 507 studies were further screened based on their titles and abstracts; 478 were excluded for a variety reasons, such as being review articles or comments, or concerning unrelated topics, leaving 29 studies that were subjected to full-text screening. Of these, three duplicated studies, two with insufficient data, and thirteen on unrelated topics were excluded. Thus, a total of 11 studies were finally subjected to qualitative and quantitative analyses [16–26].

### General characteristics and quality assessment of the studies

The general characteristics of the studies included in this meta-analysis are presented in Table 1. All included studies were published from 1997 to 2016. Sample sizes ranged from 22 to 70, including a total of 562 patients (281 for EN and 281 for PN). The mean age of patients who received EN ranged from 31.5 to 66.3 years, while that of patients who received PN ranged from 32.7 to 67.2 years. The treatment period was not significantly different between the EN and PN groups. Seven studies reported data on mortality rate, eleven provided infection and complication rates, and five provided the surgical intervention rate. Four studies provided data on MOF and four provided a mean hospitalization time.

**Table 1:** General characteristics of included in the meta-analysis

Author	Year	Age		Sample size		Sex (male/female)		Period (day)		Outcomes
		EN	PN	EN	PN	EN	PN	EN	PN	
Kalfarentzos [19]	1997	63.0 ± 10.7	67.2 ± 8.9	18	20	8/10	7/13	7	7	(1)(2)(4)(5)
Louie [20]	2005	59.0 ± 15.3	65.3 ± 18.3	10	18	6/4	9/9	13.1	14.6	(1)(2)(3)
Petrov [21]	2006	51 (42–67)	52 (41–70)	35	34	27/8	24/10	7	7	(1)(2)(3)(4)
Casas [16]	2007	61.2 ± 16.5	55.6 ± 15.6	11	11	8/3	8/3	10	10	(1)(2)(3)(4)(5)
Doley [17]	2009	38.4 ± 13.8	41.1 ± 11.3	25	25	-	-	14	14	(1)(2)(3)
Yin [24]	2012	56.4	58.7	32	33	17/15	19/14	-	-	(1)(2)
Gao [18]	2013	48.4 ± 4.1	46.2 ± 4.8	36	36	24/12	25/11	7	7	(2)(4)
Shi [22]	2014	36.1 ± 5.3	-	30	30	32/28	-	-	-	(1)(2)(3)
Tan [23]	2014	31.5 ± 1.2	32.7 ± 1.5	23	23	17/6	15/8	10	10	(2)
Zhang [26]	2015	66.3 ± 5.4	-	21	21	28/14	-	14	14	(2)(5)
Zhang [25]	2016	35.2 ± 5.5	34.8 ± 5.5	40	30	25/15	20/10	7	7	(2)(5)

(1) fatality rate (2) infection and complication (3) surgical intervention rate (4) multiple organ failure (5) Mean hospitalization time; EN, enteral nutrition; PN, parenteral nutrition.

Supplementary material 1 provides a summary of the risk of bias of the included studies, and reviews the study authors’ judgements regarding the risk for their own research. Supplementary material 2 presents these data in graph format. It is extremely difficult for clinical trials to conduct blinding. Therefore, we did classify studies into the high risk bias category on the basis of reportage of a high risk of bias with respect to the blinding of participants, study personnel, and outcome assessments. Six studies were judged to have a high risk of bias during blinding, while the level of bias was unclear for seven studies. For four studies, bias risk with respect to allocation concealment was unclear. The randomized sequence generation was adequate in all studies. No attrition or reporting biases were noted.

### Pooled results

**Figure 2:**
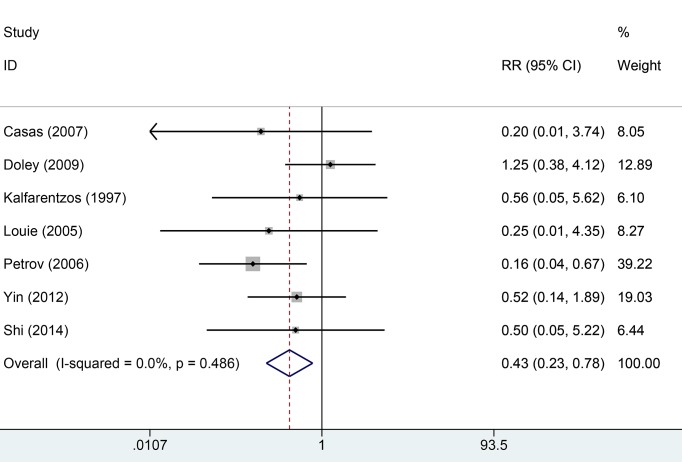
Comparison of fatality rate risk between EN and PN

**Figure 3:**
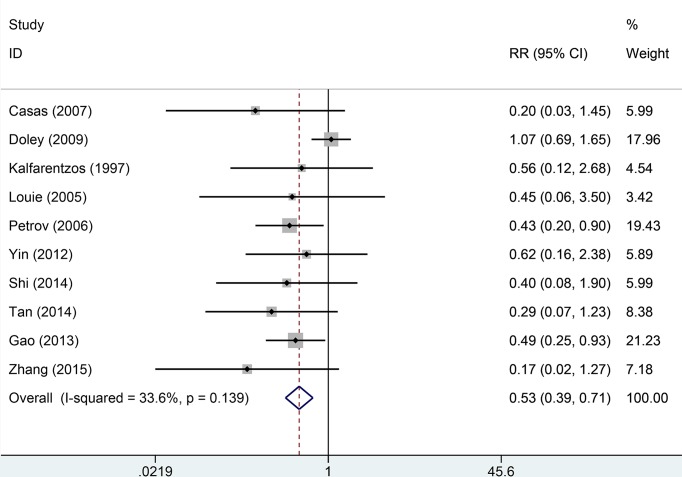
Comparison of risk of infection and complications between EN and PN

**Figure 4:**
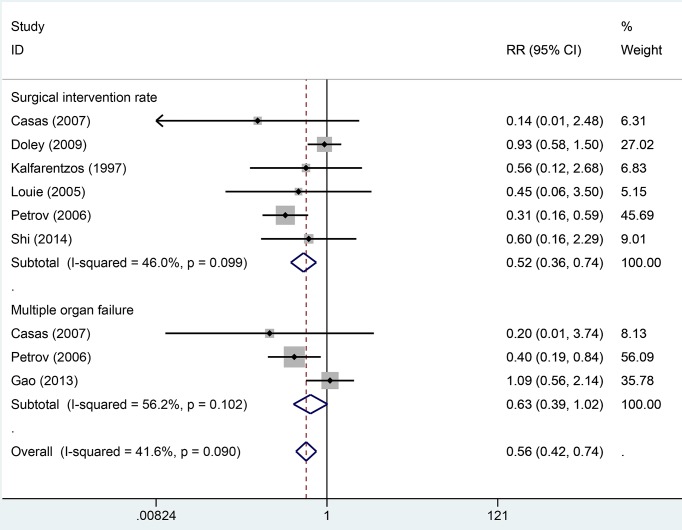
Comparison of surgical intervention and MOR between EN and PN

**Figure 5:**
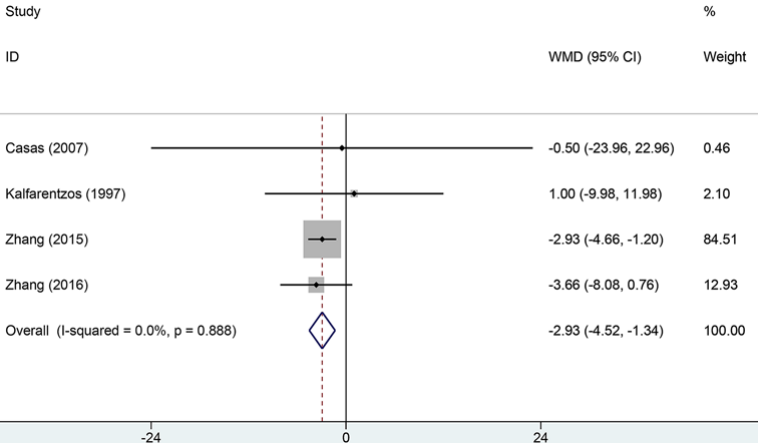
Comparison of mean hospitalization time between EN and PN

Seven studies reported mortality rate data. Heterogeneity across studies was low (*I*^2^ = 5.5%, *P*=0.486) and the fixed-effect model was used for analysis of pooled results. The results indicated that EN can significantly decrease the overall mortality rate compared with PN (RR = 0.43, 95% CI: 0.23–0.78, *P*=0.006, Figure 2). Ten studies provided information about infection and complication rates. No significant heterogeneity was found across all studies (*I*^2^ = 13.6%, *P*=0.139). We used the fixed-effect model to pool data. The results indicated that the EN groups had a lower rate of infection and complication than PN groups (RR = 0.53, 95% CI: 0.39–0.71, Figure 3). Six studies provided data on surgical intervention rates. There was low heterogeneity across all studies (*I*^2^ = 46.0%, *P*=0.099). The results of the fixed-effect model indicated that the EN groups had a lower surgical intervention rate than the PN groups (RR = 0.52, 95% CI: 0.36–0.74, *P*=0.000). There was no significant heterogeneity across three studies with respect to MOF (*I*^2^ = 56.2%, *P*=0.090). The EN groups had a similar risk of MOF to the PN groups (RR = 0.63, 95% CI: 0.39–1.02, *P*=0.059, Figure 4). Pooled results from four studies suggested that EN can significantly reduce the mean hospitalization time (MD = −2.93, 95% CI: −4.52–1.34, *P*=0.000 Figure 5); there was no significant heterogeneity across studies in this respect (*I*^2^ = 0.6%, *P*=0.888). More details of pooled results were presented in the Table 2.

**Table 2:** Summary of pooled RR in the meta-analysis

Category	Number of studies	Pooled RR/MD	95%CI (%)	*Z*	*P*	*I^2^* (%)	*P_hetero_
Fatality rate	7	0.43	0.23–0.78	2.740	0.006	5.5	0.486
Infection and complications	10	0.53	0.39–0.71	4.170	0.000	13.6	0.139
Surgical intervention rate	6	0.52	0.36–0.74	3.590	0.000	46.0	0.099
Multiple organ failure	3	0.63	0.39–1.02	1.890	0.059	56.2	0.090
Mean hospitalization time	4	−2.93	−4.52–1.34	3.610	0.000	0.6	0.888

### Sensitivity analysis and publication bias

**Figure 6:**
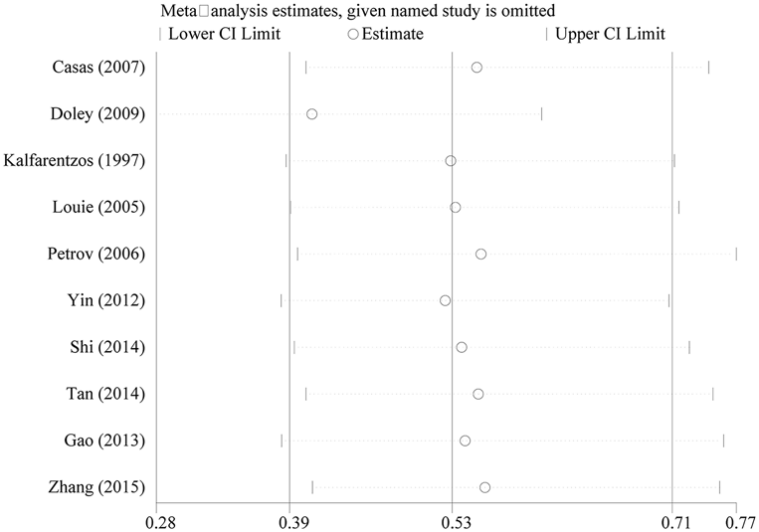
Sensitivity analysis of pooled results of infection and complications

Figure 6 presents the results of the sensitivity analysis, in which one study was excluded at a time. The results remained stable regardless of which studies were excluded, and there was no significant publication bias in any pooled analysis (*P*=0.230, 0.929, 0.602, 0.188, 0.734 for Begg’s test; and *P*=0.296, 0.236, 0.767, 0.453 for Egger’s test).

## Discussion

The current meta-analysis was based on a systematical search of the available literature and found the following: (1) EN significantly decreases the risk of death in patients with SAP compared with PN, (2) patients who received EN had a lower risk of infections and complications, a lower rate of surgical interventions, and shorter mean hospitalization time compared with patients who received PN, and (3) there was no significant difference in the rate of MOF between the EN and PN groups.

SAP is an acute pancreatitis associated with accumulation of peripancreatic fluid, pseudocysts, formation of pancreatic abscesses, and pancreatic necrosis. The severity of SAP was given by the degree of abnormality of the pancreas and its surrounding area. Balthazar et al. [27] reported that computed tomography (CT) can predict clinical outcomes by detecting abnormality of the pancreas and its surrounding area. At present, enhanced CT is the most accurate method to diagnose acute pancreatitis and its complications. Patients with SAP usually experience two phases: the first phase involves multiple organ dysfunction, sepsis, or even death, and usually lasts for approximately 7–10 days [28]. The second phase typically appears after 2 weeks, and involves risk of death secondary to pancreatic necrosis and a host inflammatory response [29]. For patients with SAP, the basal metabolic rate increases when there is an acute inflammatory response, which increases the overall energy consumption of the organism. Eighty percent of patients with SAP were found to lose more than 40 g of protein per day, resulting in a negative nitrogen balance that is not favorable with respect to recovery time [30,31]. Nutritional support is therefore of great importance in the management of SAP [32]. Previous studies also evaluated the safety and efficacy of total PN compared with total EN for patients with SAP [33–35]. However, some differences should be addressed. Previous studies have smaller sample sizes (six to nine studies) and fewer clinical parameters. The present study consisted of larger sample size (eleven studies) and more index. The latest study with nine studies reported that the duration of hospitalization was significantly shorter in the EN than PN group (mean difference, −0.59; 95% CI: −2.56–1.38), which is different from the present findings [36]. The present results indicated there was no significance in duration of hospitalization between EN and PN. Our results provided more strong evidence support.

Total gastrointestinal nutrition is the primary option for patients with SAP in early stages of the disease [37]. However, results from a recent randomized controlled trial suggests that the use of EN – that is, providing nutritional support directly through the digestive tract – is preferable to PN, because the integrity of the intestinal mucosal barrier is not compromised [38]. EN support is slowly becoming regarded as a favorable alternative treatment for SAP patients, and has been widely adopted in recent years [39]. EN support is thought to be safe for SAP patients because it does not further stimulate pancreatic function and does not have the same disadvantages as total PN. EN has been regarded as the optimal type of nutritional support for post-operative patients, especially those with severe trauma and acute pancreatitis [40]. In addition, EN can better regulate the acute phase response and maintain visceral protein metabolism, while also potentially inhibiting the toxic reaction of splenic cells [41]. Our study found that EN was better for reducing mortality, infection, and complication rates compared with PN. Our study had several strengths. First, heterogeneity across the included studies was very low, no publication bias was evident, and the she study populations were subject to the same diagnostic and treatment strategy. The baseline EN and PN data were also comparable. However, several study limitations should be acknowledged. First, the sample sizes of the included studies were small, and which may have resulted in relatively low statistical power. Furthermore, in nearly all of the studies, the patients and personnel were not blinded, which could have affected the performance and detection bias.

In conclusion, our meta-analysis found that EN, but not PN, significantly reduced the risk of mortality, and the infection and complication rates, in patients with SAP. EN support decreased the rate of MOF and surgical intervention. EN is thus recommended as the primary option for the management and treatment of patients with SAP.

## Supporting information

## Abbreviations

CI: confidence interval
CT: computed tomography
EN: enteral nutrition
MD: mean difference
MOF: multiple organ failure
PN: parenteral nutrition
RR: relative risk
SAP: severe acute pancreatitis
