# Correction: Efficacy comparisons of enteral nutrition and parenteral nutrition in patients with severe acute pancreatitis: A meta-analysis from randomized controlled trials

**DOI:** 10.1042/BSR-20181515_COR

**Published:** 2020-02-14

**Keywords:** Enteral nutrition, Parenteral nutrition, Severe acute pancreatitis, Meta-analysis, Randomized controlled trial

The authors would like to acknowledge a typographical error in the ‘Criteria for inclusion and exclusion’ section of their published article (*Bioscience Reports* (2018) **38**(6) https://doi.org/10.1042/BSR20181515) to address the statement that studies with a sample size of *n*<30 were included. The correct full statement which reflects the inclusion of studies with a sample size of *n*<20 is provided below:

“The study inclusion criteria were as follows: (1) design type: randomized controlled trial or cohort study; (2) study population: children or adults with SAP who required enteral or PN for at least 48 h; (3) intervention: enteral or PN. (4) Comparison: EN with PN. Exclusion criteria included (1) experimentation studies, comments, reviews, letters, and conferences abstracts, and (2) studies with very small sample sizes (*n*<20).”

In addition to this, the authors would like to correct the listed titles of several of the references of their published article to assist readers in finding the citation source:

22. Shi, YG and Yang, MY. (2014) Comparative analysis of enteral nutrition and parenteral nutrition in the treatment of severe acute pancreatitis. *World Health Digest* **14**, 165-165 http://www.wanfangdata.com.cn/details/detail.do?_type=perio&id=zwjkwz201414189
23. Tan, ZJ. (2014) Differences between enteral nutrition and total parenteral nutrition in nutrition and prognosis of patients with severe acute pancreatitis. *Chin J Integr Trad West Med Dig*. **22**, 619-621 http://www.wanfangdata.com.cn/details/detail.do?_type=perio&id=zgzxyjhpwzz201410018
24. Yin, L. (2012) Clinical observation of enteral nutrition combined with parenteral nutrition in the treatment of severe acute pancreatitis. *China Prac Med.* **7**, 97-98 http://kns.cnki.net/KCMS/detail/detail.aspx?FileName=ZSSA201212060&DbName=CJFQ2012
25. Zhang, QQ., Jin, ZP and Liu, LZ. (2016) Analysis of early enteral nutrition and parenteral nutrition in the treatment of severe acute pancreatitis. *J Clin Med.* **3**, 7566-7566,7567 http://www.wanfangdata.com.cn/details/detail.do?_type=perio&id=lcyydzzz201638047
26. Zhang, XJ., Xing, H., Yin, FY., Zhang, SY and Yang, G. (2015) Comparison of early enteral nutrition and total parenteral nutrition in elderly patients with severe acute pancreatitis. *J Community Med.* **13**, 45-46 http://www.wanfangdata.com.cn/details/detail.do?_type=perio&id=sqyxzz201516020

