# Supplementary material for: Efficacy comparisons of enteral nutrition and parenteral nutrition in patients with severe acute pancreatitis: a meta-analysis from randomized controlled trials
Source: Biosci Rep. 2018 Nov 16;38(6):BSR20181515. doi: 10.1042/BSR20181515 (PMC6239262; doi:10.1042/BSR20181515)
Supplement: Supplementary file 1 [file bsr20181515_Supp1.pdf]

	Random sequence generation (selection bias)	Allocation concealment (selection bias)	Blinding of participants and personnel (performance bias)	Blinding of outcome assessment (detection bias)	Incomplete outcome data (attrition bias)	Selective reporting (reporting bias)	Other bias
Casas 2007	+	+	?	?	+	+	+
Doley 2009	+	+	−	?	+	+	+
Gao 2013	+	?	?	+	+	+	+
KalFarentzos 21997	+	+	−	?	+	+	+
Louie 2006	+	+	?	−	+	+	+
Petrov 2006	+	?	?	?	+	+	+
Shi 2014	+	?	−	?	+	+	+
Tan 2014	+	?	?	?	+	+	+
Yin 2012	+	+	?	?	+	+	+
Zhang 2015	+	+	?	−	+	+	+
Zhang 2016	+	+	?	−	+	+	+

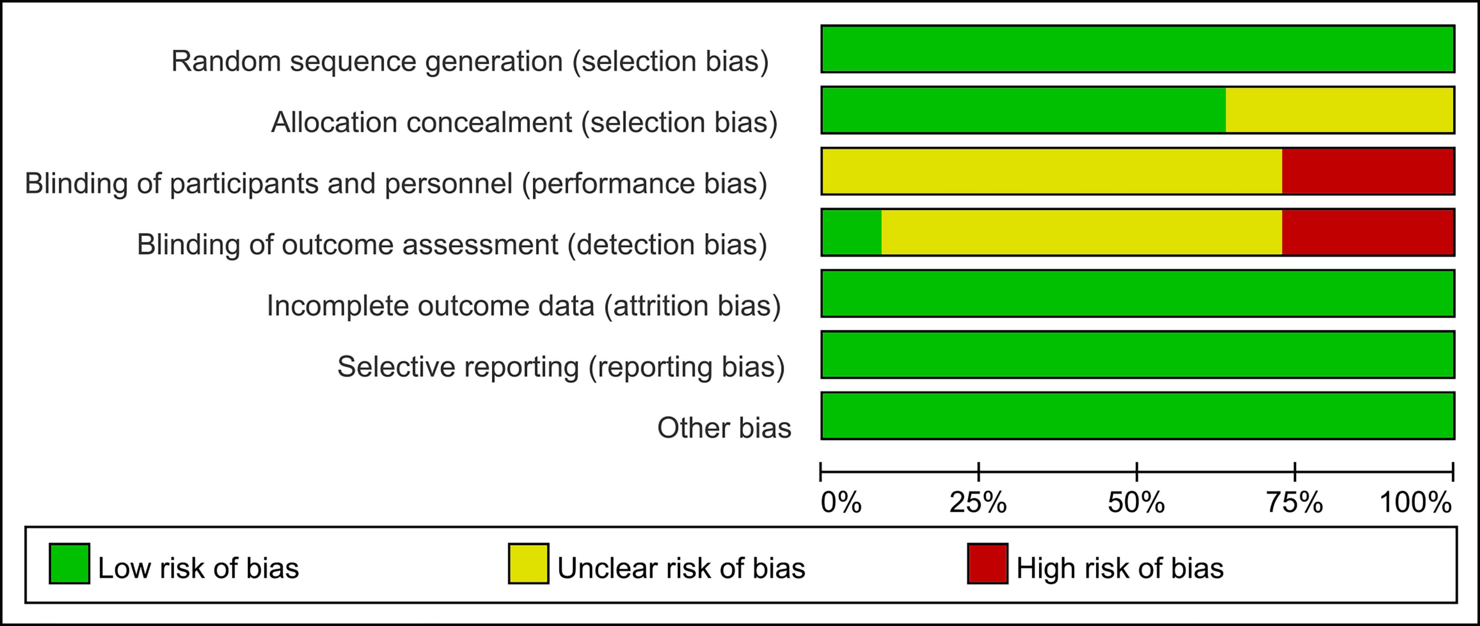
Random sequence generation (selection bias)
Allocation concealment (selection bias)
Blinding of participants and personnel (performance bias)
Blinding of outcome assessment (detection bias)
Incomplete outcome data (attrition bias)
Selective reporting (reporting bias)
Other bias
0%
25%
50%
75%
100%
Low risk of bias
Unclear risk of bias
High risk of bias
